# A Hunt for the Resistance of *Haemophilus influnezae* to Beta-Lactams

**DOI:** 10.3390/antibiotics13080761

**Published:** 2024-08-12

**Authors:** Mélanie Denizon, Eva Hong, Aude Terrade, Muhamed-Kheir Taha, Ala-Eddine Deghmane

**Affiliations:** Institut Pasteur, Université Paris Cité, Invasive Bacterial Infections Unit and National Reference Centre for Meningococci and Haemophilus influenzae, 28 Rue du Dr. Roux, CEDEX 15, 75724 Paris, Francemuhamed-kheir.taha@pasteur.fr (M.-K.T.)

**Keywords:** *Haemophilus influenzae*, beta-lactams, antibiotic resistance

## Abstract

Infections due to *Haemophilus influnezae* require prompt treatment using beta-lactam antibiotics. We used a collection of 81 isolates obtained between 1940 and 2001 from several countries. Whole genome sequencing showed the high heterogeneity of these isolates but allowed us to track the acquisition of beta-lactamase, which was first detected in 1980. Modifications of the *ftsI* gene encoding the penicillin-binding protein 3, PBP3, also involved in resistance to beta-lactams, appeared in 1991. These modifications (G490E, A502V, R517H, and N526K) were associated with resistance to amoxicillin that was not relieved by a beta-lactamase inhibitor (clavulanic acid), but the isolates retained susceptibility to third-generation cephalosporins (3GC). The modeling of the PBP3 structure suggested that these modifications may reduce the accessibility to the PBP3 active site. Other modifications appeared in 1998 and were associated with resistance to 3GC (S357N, M377I, S385T, and L389F). Modeling of the PBP3 structure suggested that they lie near the S379xN motif of the active site of PBP3. Overall resistance to amoxicillin was detected among 25 isolates (30.8%) of this collection. Resistance to sulfonamides was predicted by a genomic approach from the sequences of the *folP* gene (encoding the dihydropteroate synthase) due to difficulties in interpreting phenotypic anti-microbial testing and found in 13 isolates (16.0%). Our data suggest a slower spread of resistance to sulfonamides, which may be used for the treatment of *H. influnezae* infections. Genomic analysis may help in the prediction of antibiotic resistance, inform structure–function analysis, and guide the optimal use of antibiotics.

## 1. Introduction

The discovery and the introduction of antibiotics to clinical use spanned the second half of the 20th century. Their extensive use was thereafter associated with the emergence and spread of antimicrobial resistance (AMR). Sulfonamide drugs were first discovered and used in the 1930s [1]. The discovery of penicillin and other beta-lactams containing bioactives initiated in 1940 [1], with them being introduced to clinical use in the early 1940s. Due to their fundamental chemical structuring and auto-catalytic properties [2], beta-lactam-based antibiotics have remained effective and in use until now against a wide range of bacterial species including *Haemophilus influenzae* (Hi). This Gram-negative bacterium is responsible for local respiratory and urogenital infections and systemic infections such as bacteremia and meningitis [3,4]. *H. influenzae* can express a polysaccharide capsule whose composition defines one of the six serotypes (a to f) [5]. Non-capsulated isolates (nontypeable (NT)) are also responsible for Hi infections [3]. The introduction of a conjugate vaccine against Hib isolates drastically reduced infections due to this serotype [6,7]. However, infections due to other serotypes and NT isolates seem to persist and have increased in recent years, but no vaccines are available against these isolates [8]. Beta-lactams are still active against Hi isolates. These antibiotics interfere with the biosynthesis of the bacterial cell wall through binding of the enzymes that cross-link the peptidoglycan subunits, the penicillin-binding proteins (PBPs [9,10]). However, the extensive use of these antibiotics has driven the resistance of selected isolates, which could jeopardize the effectiveness of beta-lactams and the control of these infections, particularly in the absence of vaccines [11,12,13].

The proportion of isolates resistant to beta-lactams is increasing in several countries worldwide [11,14,15,16], and this has emerged by the acquisition of beta-lactamases, the modification of penicillin-binding protein 3 (PBP3) encoded by the *ftsI* gene, or both [11,17]. The importance of increasing resistance amongst *H. influenzae* isolates has been recognized by the WHO, and it is considered one of the bacterial priority pathogens for antimicrobial resistance [18]. Beta-lactamases are frequently of TEM-1 or ROB-1 types, which usually confer resistance to ampicillin and amoxicillin but not against third-generation cephalosporins (3GC) and are inactivated by clavulanic acid [17]. These isolates are usually referred to as beta-lactamase-positive ampicillin-resistant (BLPAR) isolates. The mutations in PBP3 involve several key residues that result in the decreased affinity of beta-lactams for PBP3 and subsequently confer resistance to ampicillin and amoxicillin (regardless of the presence of clavulanic acid), named beta-lactamase-negative ampicillin-resistant (BLNAR) isolates. Recently, mutations in additional residues of PBP3 were reported to also confer resistance to 3GC [11]. PBP3 and its corresponding isolates are classified accordingly into several groups [11,17,19]. The BLNAR isolates have been described since 1980. A significant increase in these isolates was documented worldwide, and wide variations exist in different countries [20,21,22], but the molecular mechanisms and the evolution of *ftsI* sequences for isolates spanning the period before and after the use of beta-lactams have not been documented. We, therefore, aimed in this retrospective study to analyze the mechanisms of β-lactam and cotrimoxazole resistance amongst a collection of Hi isolates that spanned the period before and after the use of beta-lactams and their correlation with antibiotic susceptibility profiles, with special emphasis on BLNAR isolates.

## 2. Results

### 2.1. Characteristics of the Historic Collection of H. influenzae Isolates

**Figure 1:**
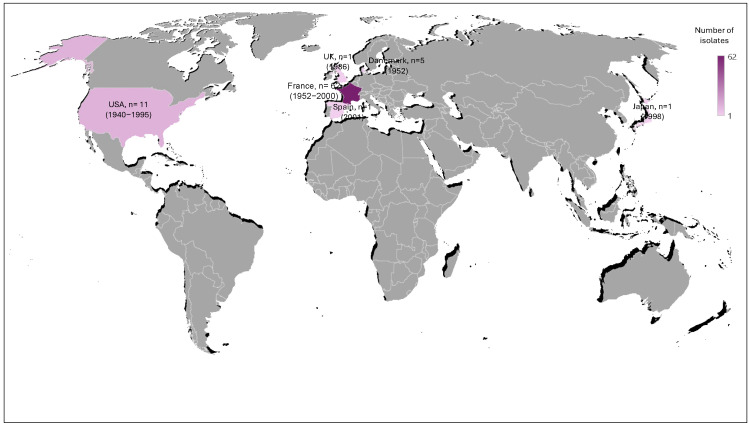
Geographical distribution of *H. infuenzae* isolates used in this study. The number of isolates is indicated for each country and the period of isolation is indicated between parentheses. The color intensity is proportional to the number of isolates.

We first screened a historical collection of the Institut Pasteur that is composed of 81 isolates that were isolated between 1940 and 2001 from several countries including France, Denmark, Japan, Spain, the UK, and the USA. The geographical distribution and period of isolation of these strains are summarized in Figure 1. For 54 isolates, the isolation site was known: respiratory sites including otitis and conjunctivitis (n = 34), CSF (n = 7), urogenital sites (n = 6), blood (n = 4), placenta (n = 2), and peritoneal liquid (n = 1) (Supplementary Table S1). Most of these isolates were NT (non-typeable) (75.3%), while serotype b and the other serotypes (a, c, d, e, f) represented 13.6% and 11.1%, respectively (Supplementary Table S1).

We next performed antibiotic susceptibility testing for the 81 isolates for amoxicillin, amoxicillin/clavulanic acid, cefotaxime, and cotrimoxazole (trimethoprim/sulfamethoxazole). The isolates were also tested for the production of beta-lactamases (Table 1). We detected 14 beta-lactamase-producing isolates (2 Hib and 12 NT isolates). The earliest beta-lactamase-producing isolate was from the year 1980 in the USA, with the first French isolate in the year 1985 (Supplementary Table S1). As expected, all these 14 (17.2%) isolates were resistant to amoxicillin (MIC range: 3–256 mg/L). Among these beta-lactamase-positive amoxicillin-resistant (BLPAR) isolates, 10 were susceptible to the combination of amoxicillin/clavulanic acid (MIC range: 0.125–1.0 mg/L) and 4 remained resistant to amoxicillin/clavulanic acid (MIC 3 mg/L), despite the presence of the inhibitor of beta-lactamase. All these beta-lactamase-producing isolates were susceptible to cefotaxime (MIC range: 0.008–0.064 mg/L). Among the other 67 beta-lactamase-negative isolates, 56 were susceptible to amoxicillin, amoxicillin/clavulanic acid, and cefotaxime, while 10 isolates were resistant to amoxicillin and amoxicillin/clavulanic acid but remained susceptible to cefotaxime. The earliest isolate in this collection of beta-lactamase-negative amoxicillin resistance (BLNAR) was detected in the year 1991 in France (Supplementary Table S1, ID 15153). Finally, one isolate (Supplementary Table S1, ID 15154) was resistant to amoxicillin, amoxicillin/clavulanic acid, and cefotaxime. This isolate was detected in the year 1998 in Japan. The data indicate that 11 isolates (13.6%) of this collection were therefore BLNAR. The overall resistance to amoxicillin (both BLNAR and BLPAR) was detected for 25 isolates and accounted for 30.8% of this collection of isolates.

### 2.2. Genetic Characteristics of the Historic Collection of H. influenzae Isolates

**Figure 2:**
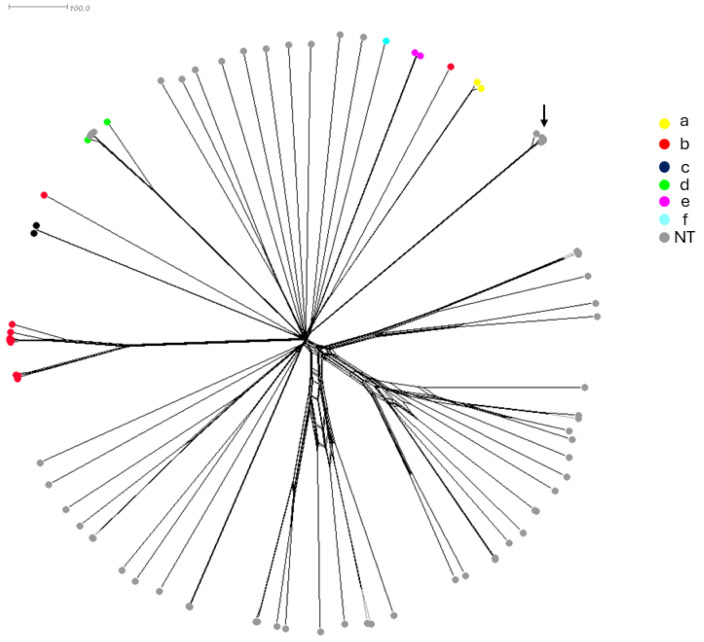
A neighbor network based on the cgMLST allelic profiles of the 81 isolates in this study. Individual isolates are represented by circles and the color of the circle indicates the serotype of the corresponding isolate. The five *H. quentini* isolates were clustered together (arrow).

Whole genome sequences were also performed on all 81 isolates and compared by a gene-by-gene approach using the 1037 gene of core-genome MLST (cgMLST) available on the PUBMLST.org database (accessed on 2 August 2023) [23]. The genome-based prediction of the capsule correlated with the PCR-based serotyping. The cgMLST-based tree showed the highly heterogeneous structure of the isolates, particularly the NT isolates, as the serotypeable isolates were clustered together according to each serotype (Figure 1). Notably, four NT isolates (Supplementary Table S1 IDs 15135, 15137, 15160, and 15165) clustered with the serotype d-specific branch and belonged to the most detected clonal complex within this serotype (CC10). These isolates may have lost their capsule locus. Conversely, two serotype b isolates (Supplementary Table S1 IDs 15099 and 15171) were clustered among NT isolates, suggesting acquisition of the capsule locus. These results point out the genetic diversity among Hi isolates. Five of the eighty-one isolates (Supplementary Table S1 IDs 15098, 15107, 15116, 15131, and 15132) were identified by WGS as *Haemophilus quentini* and were clustered together but not separated from the other seventy-six isolates. These five isolates were all from France between 1984 and 1986 (Figure 2). These isolates were kept in the analysis and were from both invasive and non-invasive infections (Supplementary Table S1).

### 2.3. Characteristics of Genes Involved in Antibiotic Resistance

All 14 beta-lactamase-producing isolates harbored beta-lactamase-encoding genes that were most frequently (n = 13, 93%) the class A broad-spectrum beta-lactamase TEM-1, and only one isolate harbored ROB-1 beta-lactamase.

**Figure 3:**
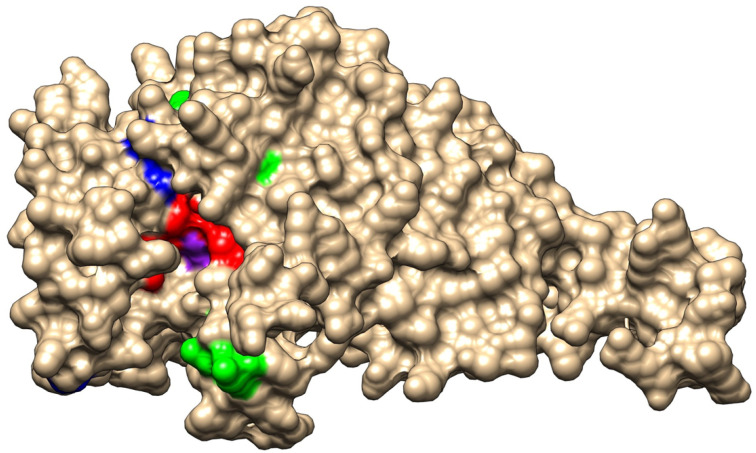
Representation of PBP3 modeled structure. A surface view of the structure in the representation of wild-type PBP3. The three motifs of the active site pocket (S327xxK, S379xN, and K512TG) are shown in red and active S327 is shown in purple). The critical residues for the resistance to amoxicillin (G490 A502, R517, and N526) are shown in green. The critical residues for resistance to amoxicillin (S357, M377, S385, and L389) are shown in blue, particularly M377, which lies very close to the active site pocket.

We also extracted from the WGS data alleles of the gene *ftsI*, encoding PBP3 and involved in resistance to beta-lactams, which were studied extensively for mutations correlated to resistance to beta-lactams [11]. Among the 81 isolates of this collection, we identified 39 distinct *ftsI* alleles encoding 21 distinct 207 aa-domains of PBP3 involved in resistance to beta-lactams [11]. PBP3 encoded by the *ftsI* alleles was distributed in the four groups that were previously described [11]: group 1, with a wild-type sequence in the region of *ftsI* involved in resistance to beta-lactams, but can harbor a polymorphic site outside this region (52 isolates harboring 17 distinct alleles); group 2, with polymorphic sites within the region but no critical mutations (14 isolates harboring 8 distinct alleles); group 3, with mutations in the region conferring resistance to ampicillin, amoxicillin, and amoxicillin/clavulanic acid at the critical positions (G490E, A502V, R517H, and N526K) but no mutation conferring resistance to third-generation cephalosporins (14 isolates harboring 13 distinct alleles); and group 4, with mutations conferring resistance to third-generation cephalosporins (S357N, M377I, S385T, and L389F) in addition to mutations of group 3 (1 isolate and 1 allele). These critical residues that are involved in resistance to beta-lactams are located near the active site of PBP3 (S327xxK, S379xN, and K512TG motifs) (Figure 3). Of interest, the residues (G490E, A502V, R517H, and N526K) involved in resistance to ampicillin, amoxicillin, and amoxicillin/clavulanic acid are located at the entrance of the pocket of the active site, while most of the residues (S357N, M377I, S385T, and L389F) that are involved in resistance to third-generation cephalosporines are located in the vicinity of the active site and particularly close to the S379xN motif and the M377I mutation (Figure 3).

**Figure 4:**
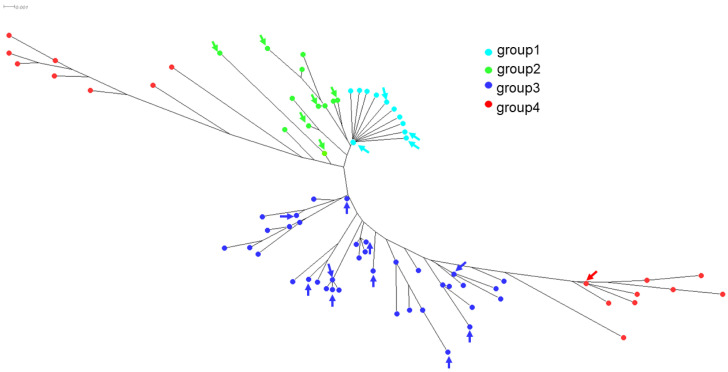
Phylogenetic tree of *ftsI* based on multiple alignments of amino acid sequences deduced by the DNA sequences of the 160 unique PBP3 amino acid sequences. The tree was visualized by SplitsTree4 as described in the Materials Methods section. The four groups of PBP3 were defined according to our previous work [11]. Group 1 (wild-type sequences) and group 2 (no critical mutations) corresponded to amoxicillin-susceptible isolates, group 3 corresponded to mutations (G490E A502V, R517H, and N526K) corresponding to amoxicillin-resistant isolates, and group 4 corresponded to mutations conferring resistance to amoxicillin and 3rd-generation cephalosporins (S357N, M377I, S385T, and L389F). The 21 distinct 207 aa-domains of PBP3 involved in the resistance to beta-lactams of the current study are indicated by arrows in each of the four groups.

The 21 predicted amino acid sequences of the 207 aa domains were aligned together with the identified *ftsI* allele sequences in *H. influenzae* that we have previously reported [11]. A total of 160 unique amino acid sequences were therefore aligned and the corresponding phylogenetic tree was visualized using SplitsTree (version 4.14.6). The 21 PBP3 sequences identified in this study were localized on several regions of the phylogenetic tree within the four already identified groups of PBP3 (Figure 4). Most of the 21 PBP3 predicted sequences belonged to groups 1 and 2, which corresponded to beta-lactam-susceptible isolates. Several alleles were represented by several isolates with allele *ftsI10* (group 1), which was the most frequently encountered among the 81 isolates of this study (17 isolates; 21.0%). *ftsI* alleles of group 3 were detected (n = 13) and corresponded to 14 isolates, among which, 10 isolates were beta-lactamase-negative and resistant to amoxicillin and amoxicillin+clavulanic acid but susceptible to cefotaxim. Four isolates were both beta-lactamase-positive and harbored alterations in PBP3 (Supplementary Table S1 IDs 15143, 15144, 15151, and 15152). One isolate (Supplementary Table S1 ID 15154) harbored a *ftsI* allele of group 4 (*ftsI40*) and corresponded to the unique isolate that was resistant to amoxicillin, amoxicillin + clavulanic acid, and cefotaxime. This isolate was also resistant to ceftriaxone (Supplementary Table S1).

Overall, 10 isolates harbored beta-lactamases with no critical PBP3 mutations, 11 isolates were beta-lactamase-negative but with critical PBP3 mutations, and 4 isolates harbored both traits. A complete correlation between phenotypic antibiotic susceptibility testing and the molecular data on the presence of beta-lactam-encoding genes and from *ftsI* sequences was noticed (Table 1 and Supplementary Table S1).

Resistance to cotrimoxazole was determined from WGS data based on the presence of a length variable insertion in the *folP* gene (*Haem1634*) encoding the dihydropteroate synthase that was reported to confer resistance [24]. This insertion was detected in 13 isolates (Supplementary Table S1). These 13 isolates were tested by phenotypic antimicrobial testing (antibiogram) according to the recommendation of the EUCAST [25]. The MIC of cotrimoxazole ranged between 0.008 and 3 mg/L for the 13 isolates. Five of these thirteen isolates (Supplementary Table S1 IDs 15139, 15146, 15147, 15148, and 15149) were resistant to cotrimoxazole by disc diffusion antibiogram and three of them (IDs 15139, 15146, and 15147) were also found to be resistant by gradient E-tests (Supplementary Table S1). The 13 isolates harbored several lengths of the insertion and several *folp* alleles (Supplementary Table S1).

## 3. Discussion

This work allowed for the analysis of a collection of historical *H. influenzae* from the period of 1940–2000 of the 20th century, spanning several countries since the introduction of sulfonamides and penicillin for the emergence of antibiotic-resistant bacteria. Although the number of isolates was limited to 81, valuable information was obtained on the phenotypic and genotypic diversity of the isolates as well as phenotypic and genotypic data on their resistance to antibiotics and particularly to beta-lactams. The collection was phenotypically diverse and all *H. influnezae* serotypes were presented as well as non-typeable isolates. The identification of five *H. quentini* isolates that were misidentified as *H. influnezae* underlines the need for molecular data for the reliable identification of species in the *Haemophilus* genus. However, we kept these isolates in our analysis as *H. quentini* is highly related to *H. influenzae* and was characterized in the 1990s as a cryptic genospecies of *Haemophilus* [26]. The 81 isolates were from different types of infection including respiratory and genital infections as well as invasive infections (meningitis and bacteremia) (Supplementary Table S1). Resistance to beta-lactams by the acquisition of a beta-lactamase seems to have appeared at least since 1980 in the USA and 1985 in France. As expected, TEM-1 beta-lactamase was the most frequently detected among the isolates in this study. These findings are in general agreement with the larger prevalence of TEM-1 than ROB-1 among β-lactamase-positive *H. influenzae* strains worldwide [27]. These isolates remained susceptible to the association of amoxicillin with clavulanic acid. The appearance of modifications in the *ftsI* gene encoding PBP3 in BLNAR isolates was reported later in France in 1991 and seems to have increased since then. Amoxicillin-resistant isolates accounted for about two-thirds of Hi non-typeable isolates between 2017 and 2021 in France [28]. This delay agrees with the involvement of this protein in essential functions for cell division [29] and is in favor of the biological cost of these modifications in invasive isolates [11]. Corroboratively, a significantly higher proportion of BLNAR was reported among respiratory isolates than among invasive isolates [11,28]. The covalent attachment of β-lactams to PBP3 occurs via acylation of the conserved S327xxK motif, creating a steric barrier that prevents PBP3 from being released [30,31]. The modifications of PBP3 that seem to confer the BLNAR phenotype involve mainly residues G490E, A502V, R517H, and N526K, which lie at the entrance to the active site pocket and may disrupt the arrangement of residues in the active site region and therefore prevent the accessibility of the active site to amoxicillin, but the corresponding isolates are expected to retain the sensitivity to third-generation cephalosporins, which were reported to show better protein–ligand binding affinity than amoxicillin as cephalosporins express lower binding energy to PBPs than penicillin derivatives [32]. However, other modifications in PBP3 arose and were associated with resistance to cephalosporines (S357N, M377I, S385T, and L389F). These modifications lie very close to the active site within the alpha helices 4 and 5 close to the loop between these two helices, where lies the SxN motif of the active site of PBPs [33]. The impact of these modifications is expected to be more drastic on the binding of both cephalosporins and penicillin derivatives to PBP3. These isolates are therefore resistant to both types of beta-lactams. Only one allele (*ftsI40*) harboring cephalosporins-associated modifications was detected in one isolate (Japan 1998) of the collection of this study (Supplementary Table S1). Such isolates may have been selected later than the isolates harboring the amoxicillin-associated modifications [11].

Surprisingly, a lower percentage of isolates were resistant to cotrimoxazole (trimethoprim/sulfamethoxazole) compared to beta-lactams (16.0% versus 35.8%, respectively). These historical data suggest a slower rate of spread of resistance to cotrimoxazole compared to amoxicillin resistance, despite the early introduction of sulfonamide drugs. This susceptibility to cotrimoxazole is in contrast to the rapid acquisition of resistance to sulfonamides in *Neisseria gonorrhoeae* in the 1940s upon their introduction and use for the treatment of gonorrhea [34]. Moreover, moderate proportions of resistance to cotrimoxazole among recent *H. influenzae* did not exceed one-third of the respiratory isolates in France between 2017 and 2021 [28]. Resistance to cotrimoxazole was characterized in this work using sequence analysis of the *folP* gene encoding dihydropteroate synthase, but the correlation with phenotypic testing was poor. This observation may suggest that mutations in the *folP* gene for a resistant phenotype may be of low penetrance. Indeed, Hi isolates harboring different mutations in *folP* expressed variable levels of resistance to cotrimoxazole [24]. Moreover, phenotypic testing of resistance to cotrimoxazole requires the use of media prepared with defibrinated and lysed horse blood as horse erythrocytes contain thymidine phosphorylase, which inactivates thymidine to thymine, which, unlike thymidine, does not interfere with the activity of cotrimoxazole. All these considerations underscore the need to use molecular analysis in antibiotic susceptibility testing.

The higher *H. influnezae* susceptibility to cotrimoxazole than to beta-lactams is relevant. About 62% of Hi isolates that were resistant to amoxicillin were reported to be susceptible to cotrimoxazole [28]. This observation may advocate for the use of cotrimoxazole instead of amoxicillin or an amoxicillin/clavulanic acid combination as the first-line antibiotic treatment in respiratory infections due to Hi isolates such as acute otitis media, for which Hi is increasingly encountered in recent years [28].

## 4. Materials and Methods

### 4.1. Isolates and Analysis

This was a retrospective study that used isolates from the collection of the Institut Pasteur, Paris, France, and included 81 isolates that were collected between 1940 and 2001 in France (n = 62), the USA (n = 11), Denmark (n = 5), the UK (n = 1), Spain (n = 1), and Japan (n = 1). The country, year, and source of isolation (when available) for each isolate are described in Supplementary Table S1. These isolates were recovered at the French National Reference Center for meningococci and *H. influenzae* (NRCMHi). The NRCMHi is a laboratory designated under the authority of the Ministry of Health for the microbiological surveillance of meningococcal and *H. influenzae* infections. The NRCMHi receives and characterizes Hi isolates and provides analyses of several epidemiological aspects of Hi infections including the surveillance of AMR. The procedure for collecting samples and information was submitted and approved by the CNIL N°1475242/2011 (Commission Nationale de l’Informatique et des Libertés). The isolates were cultured on chocolate agar medium (bioMérieux, Marcy-l’Etoile, France) and serotypes were determined using a PCR-based approach as previously described [35]. A typeable isolate was identified as positive by PCR for one of the serotype-specific genes. Non-typeable isolates were identified as negative for *bexD* and all of the serotype-specific PCRs [35].

The AMR testing was performed on the isolates that were recovered by culture at the NRCMHi using gradient tests (E-test, bioMérieux, Marcy-l’Etoile, France). We tested amoxicillin (MIC range: 0.016–256 µg/mL), amoxicillin/acid clavulanic (MIC range: 0.016–256 µg/mL with clavulanic acid at constant concentration of 2 µg/mL), cefotaxime (a third degeneration cephalosporin, MIC range: 0.002–32 µg/mL), and cotrimoxazole (trimethoprim/sulfamethoxazole, 1:19 ratio, MIC range: 0.002–32 µg/mL). When indicated, cotrimoxazole (1:19 ratio) susceptibility was also tested by disc diffusion (Oxoid, Dardilly, France). Commercial MH-F agar medium was used based on the European Committee on Antimicrobial Susceptibility Testing (EUCAST) guidelines. β-lactamase production was measured using the cefinase disc method (bioMérieux, Marcy-l’Etoile, France). The EUCAST for the antimicrobial susceptibility breakpoints of the minimum inhibitory concentration (MIC) of antibiotics was adopted as the criteria for interpreting drug susceptibility [25].

### 4.2. Molecular Analysis

Whole genome sequencing (WGS) was performed as previously described [11]. Briefly, genome sequencing data for each isolate DNA were extracted from plated isolates by using the MagNA Pure 96 system (Roche Molecular System, Pleasanton, CA, USA). Library preparation was performed with the Nextera^®^ XT DNA library Preparation Kit (Illumina, San Diego, CA, USA) and whole genome sequencing (WGS) data were generated using Illumina^®^ technology (NextSeq 500, Illumina) (San Diego, CA, USA) with paired-end strands of 150 bp and a sequencing depth of 50×. All de novo assemblies were performed with SPAdes (CAB, St. Petersburg State University, St. Petersburg, Russia). The genome assemblies for all 81 isolates used in this study have been loaded onto the https://pubmlst.org database (accessed on 2 August 2023) and can be retrieved on the basis of the ID numbers listed in Supplementary Table S1. Genomic analyses were performed using the tools available on the BIGSdb platform including multilocus sequence typing (MLST) and core-genome MLST. The sequences of a 621-bp fragment of the *ftsI* gene (encoding PBP3), corresponding to nucleotides 977–1597 relative to the *ftsI* start codon, were extracted from WGS data. *ftsI* sequences were aligned using Multiple Sequence Alignment by CLUSTALW and phylogenetic networks were generated using SplitsTree (version 4.14.6 https://uni-tuebingen.de/fakultaeten/mathematisch-naturwissenschaftliche-fakultaet/fachbereiche/informatik/lehrstuehle/algorithms-in-bioinformatics/software/splitstree/, accessed on 30 March 2024) with default parameters [36].

### 4.3. Structure–Function Analysis

The whole sequences of *ftsI* (*Haem1263*) genes were translated and then loaded and modeled on Phyre2 databases (www.sbg.bio.ic.ac.uk/phyre2/ accessed on 15 April 2024) using Protein Homology/analogY Recognition Engine V 2.0. The different models were analyzed and visualized using Chimera 1.18 software developed by the University of California at San Francisco [37].

### 4.4. Statistical Analysis

Statistical analyses were performed using GraphPad Prism 5.01 software, San Diego, CA, USA, (www.graphpad.com accessed on 28 March 2024). A Bonferroni correction was applied by adjusting the α risk to the number of comparisons performed according to the formula α = 0.05/n, where n is the number of comparisons performed.

## 5. Conclusions

The global health community is facing a daunting challenge in the field of antibiotic resistance, with *H. influenzae* at the forefront. During the period that spans before and after the use of beta-lactams, resistance to beta-lactams emerged in 1980 and was mediated by the acquisition of beta-lactamases. Resistance to beta-lactams evolved thereafter to include non-enzymatic mechanisms comprising modifications in critical residues of PBP3. These modifications, which first appeared in 1991, conferred resistance to amoxicillin, but, since 1998, other critical changes in PBP3 were associated with resistance to 3GC. Resistance to sulfonamides seems to evolve slowly, making these antibiotics an alternative for the treatment of *H. influnezae* infections. The continuous monitoring of antibiotic resistance and the molecular evolution of *H*. *influenzae* is extremely necessary to respond to new epidemiology trends and can guide structure–function analysis to inform the optimal use of antibiotics.

## Figures and Tables

**Table 1:** Beta-lactam susceptibility testing of the historical collection of *H. influenzae*.

N°	Beta-Lactamase	Amoxicillin: Phenotype * (Range MIC mg/L)	Amoxicillin/Clavulanic Acid: Phenotype (Range MIC mg/L)	Cefotaxime: Phenotype (Range MIC mg/L)
56	Negative	S (0.25–1)	S (0.25–1)	S (0.004–0.125)
10	Negative	R (3–256)	R (3–256)	S (0.032–0.125)
1	Negative	R (256)	R (256)	R (4)
10	Positive	R (3–256)	S (0.125–1)	S (0.008–0.023)
4	Positive	R (64–256)	R (3)	S (0.032–0.064)

* Phenotypes: S—susceptible, R—resistant.

## Data Availability

The genomic data (FASTA files) for *H. influenzae* can be retrieved from the https://pubmlst.org site (accessed on 2 August 2023) by filtering by country (France) and ID number.

## Supplementary Materials

The following supporting information can be downloaded at https://www.mdpi.com/article/10.3390/antibiotics13080761/s1: Supplementary Table S1: Characteristics of *H. influenzae* isolates used in this study.
